# Detection of *Elizabethkingia* spp. in *Culicoides* Biting Midges, Australia

**DOI:** 10.3201/eid2308.161565

**Published:** 2017-08

**Authors:** Peter T. Mee, Stacey E. Lynch, Peter J. Walker, Lorna Melville, Jean-Bernard Duchemin

**Affiliations:** The University of Melbourne, Parkville, Victoria, Australia (P.T. Mee); Agriculture Victoria, AgriBio, Bundoora, Victoria, Australia (P.T. Mee, S.E. Lynch);; Commonwealth Scientific Industrial Research Organization, Geelong, Victoria, Australia (P.T. Mee, P.J. Walker. J.-B. Duchemin);; University of Queensland, St Lucia, Queensland, Australia (P.J. Walker);; Department of Primary Industry and Fisheries, Berrimah, Northern Territory, Australia (L. Melville)

**Keywords:** Elizabethkingia, Culicoides, biting midge, Culicoides brevitarsis, mosquito, Australia, bacteria

## Abstract

The bacterial pathogen *Elizabethkingia* is known to exist in certain species of mosquito but was unknown in other arthropods. We report the detection and identification of *Elizabethkingia* in species of *Culicoides* biting midge in Australia, raising the possibility of bacterial transmission via this species.

Bacteria in the genus *Elizabethkingia* (formerly *Chryseobacterium* or *Flavobacterium*) are gram-negative bacilli that occur globally in water sources including rivers, reservoirs, and soils. In recent years, 3 medically important species in this genus, *E. meningoseptica*, *E. anophelis,* and *E. miricola*, have been recognized as the cause of emerging nosocomial infections, neonatal sepsis, and infections in immunocompromised persons. Outbreaks and infections have occurred globally, with cases reported in the Central African Republic; Singapore; Hong Kong, China (*1*); India; Australia; and the United States. Infection by any of the 3 species can cause septicemia, with a recorded mortality rate of 23.5% (*1**–**3*).

Originally, *E*. *meningoseptica* was thought to be the causative agent of most *Elizabethkingia* infections. However, *E*. *anophelis* has recently been implicated as the more likely cause and as the primary species associated with bacteremia (*1*). Isolated from *Anopheles gambiae* mosquitoes in 2011 (*4*), *E*. *anophelis* has a relatively high occurrence (68%) in field-collected mosquitoes (*5*) and has been identified in *Aedes aegypti* and *An. stephensi* mosquitoes (*6*,*7*), with transmission between mosquitoes by vertical, horizontal, and transstadial modes (*6*,*7*). Occurrence of *Elizabethkingia* in other arthropods has not been reported.

*Culicoides* biting midges are classified in the family Ceratopogonidae in the order Diptera. These arthropods are found around the world and are capable of transmitting pathogens (mainly viral or filarial but also bacterial) affecting birds, livestock, and humans. In Australia, there are 78 described and 61 undescribed species of *Culicoides* midges. We investigated the presence of *Elizabethkingia* in *Culicoides* midges in Australia.

In summer 2013, we collected 66 *Culicoides* individuals in Australia from 3 locations (Table) using light traps: 24 *C. victoriae*, 21 *C. multimaculatus*, and 21 *C. brevitarsis*. We examined them for the presence of *Elizabethkingia* using 16S rRNA amplicon sequencing. *Culicoides* were collected from 3 locations (Table) using light traps. The midges were identified to species morphologically from homogenized females at CSIRO, Geelong, Victoria, Australia, before DNA extraction using a QIAGEN blood and tissue kit (QIAGEN, Valencia, California, USA). *Culicoides* species identification was confirmed by sequencing the COI gene (*8*). 

**Table:** Collection locations and sequencing results for study of *Elizabethkingia* in *Culicoides* biting midges, Australia*

*Culicoides* species	Collection location	Collection coordinates	% Infected with *Elizabethkingia* (no. infected/no. collected)	Average no. *Elizabethkingia* reads per individual (95% CI)	Average % *Elizabethkingia* reads to bacterial reads (95% CI)
*C. brevitarsis*	Beatrice Hill, Northern Territory	12°39′S, 131°19′E	71 (15/21)	1,419 (724–2,113)	0.075 (0.05–0.1)
*C. multimaculatus*	Lake Wellington, Victoria	38°23′S, 147°21′E	0 (0/21)	NA	NA
*C. victoriae*	Grampians, Victoria	38°08′S, 142°12′E	0 (0/24)	NA	NA

*NA, not applicable

We amplified the hypervariable region V3-V4 of the 16S rRNA (422 bp) using PCR primers (S-D-Bact-0341-b-S-17/S-D-Bact-0785-a-A-21) (*9*), following a modified 16S MiSeq protocol and barcoded samples before sequencing on a MiSeq (Illumina, Victoria, British Columbia, Canada). Two negative controls were included throughout the sequencing methodology to ensure no contamination. We analyzed data using the Quantitative Insights into Microbial Ecology pipeline (*10*) with reference to Greengenes database. To confirm *Elizabethkingia* species, we used primers forward 5′-ATCTTCATGGAAGGAGAGC-3′ and reverse 5′-GTACCAACACTTACCCCTAA-3′ to amplify 670 bp of the gene encoding the subunit B protein of the DNA gyrase (*gyrB*). Amplicons were purified and then sequenced using a capillary Sanger method.

Sequencing analysis identified a total of 2,717,401 operational taxonomic units across the 66 samples, after all quality trimming procedures and removal of chimeras. Based on the 16S rRNA (GenBank accession no. KX870017) amplicon sequence (>280 times coverage), we determined that *Elizabethkingia* infected only *C*. *brevitarsis* midges (Table). We did not detect *Elizabethkingia* in negative control samples. 

*Elizabethkingia* sequences had 100% nucleotide identity among the 15 *C. brevitarsis* individuals and high sequence identity to *E*. *anophelis* (99.05%–99.29%), *E*. *meningoseptica* (98.34%–100%), and *E*. *miricola* (99.76%) based on sequences obtained from GenBank. However, we were unable to identify the exact strain based on 16S rRNA alone. We successfully amplified *Elizabethkingia gyrB* (GenBank accession no. KX870018) and concatenated sequences with the 16S rRNA region, forming a 1,072-bp amplicon. The phylogenetic analysis confirmed *E*. *anophelis* as the closest to the species isolated from *C*. *brevitarsis*, with 95.8% identity across the 2 amplified gene regions. 

The *C. brevitarsis* midge is a known vector of several pathogenic viruses in livestock of Australia. Of the many diverse *Culicoides* midge species, some transmit human pathogens; the close association of some species with humans suggests a need for a more detailed study.

Although we did not detect *Elizabethkingia* in *C*. *victoriae* or *C*. *multimaculatus* midges, this finding may be a result of differences in climate or ecosystem. Unlike mosquitoes that breed in water, the *C*. *brevitarsis* midge uses cow dung, so it is potentially isolated from environmental contamination of *Elizabethkingia*. Because *Culicoides* midges are widespread and can be displaced great distances by wind currents, the potential for them to transport the bacterium warrants further investigation. 

The nature of the association between insects and *Elizabethkingia* is unknown. Mosquitoes have been reported to harbor *Elizabethkingia*, but it is unknown if they act as vectors or simply serve as reservoirs, symbionts, or environmental markers. One study investigating the possible role of mosquitoes as vectors of *Elizabethkingia* suggests that they act as reservoirs (*3*). Nevertheless, while the role of insects as vectors of infection remains unclear, the identification of *Elizabethkingia* in *Culicoides* midges is relevant to public health. The *gyrB* primers developed in this study allow more accurate diagnosis of *Elizabethkingia* species than a single gene classification.
